# Cross-talk between cuproptosis and ferroptosis regulators defines the tumor microenvironment for the prediction of prognosis and therapies in lung adenocarcinoma

**DOI:** 10.3389/fimmu.2022.1029092

**Published:** 2023-01-17

**Authors:** Yefeng Shen, Deyu Li, Qiong Liang, Mengsi Yang, Youguang Pan, Hui Li

**Affiliations:** ^1^ Institute for Pathology, University Hospital of Cologne, Cologne, Germany; ^2^ Department of Thoracic Surgery, Zhujiang Hospital, Southern Medical University, Guangzhou, China; ^3^ Department of Medical Oncology, Provincial Clinical College, Fujian Medical University, Fujian Provincial Hospital, Fuzhou, China; ^4^ Department of Respiratory Disease, The Affiliated Hospital of Youjiang Medical University for Nationalities, Baise, China; ^5^ Thoracic and Cardiovascular Surgery, The Third Affiliated Hospital of Guangzhou Medical University, Guangzhou, China

**Keywords:** cuproptosis, ferroptosis, CuFescore, tumor microenvironment, prognosis, chemotherapy, immunotherapy

## Abstract

Cuproptosis, a newly identified form of programmed cell death, plays vital roles in tumorigenesis. However, the interconnectivity of cuproptosis and ferroptosis is poorly understood. In our study, we explored genomic alterations in 1162 lung adenocarcinoma (LUAD) samples from The Cancer Genome Atlas (TCGA) and the Gene Expression Omnibus (GEO) cohort to comprehensively evaluate the cuproptosis regulators. We systematically performed a pancancer genomic analysis by depicting the molecular correlations between the cuproptosis and ferroptosis regulators in 33 cancer types, indicating cross-talk between cuproptosis and ferroptosis regulators at the multiomic level. We successfully identified three distinct clusters based on cuproptosis and ferroptosis regulators, termed CuFeclusters, as well as the three distinct cuproptosis/ferroptosis gene subsets. The tumor microenvironment cell-infiltrating characteristics of three CuFeclusters were highly consistent with the three immune phenotypes of tumors. Furthermore, a CuFescore was constructed and validated to predict the cuproptosis/ferroptosis pathways in individuals and the response to chemotherapeutic drugs and immunotherapy. The CuFescore was significantly associated with the expression of miRNA and the regulation of post-transcription. Thus, our research established an applied scoring scheme, based on the regulators of cuproptosis/ferroptosis to identify LUAD patients who are candidates for immunotherapy and to predict patient sensitivity to chemotherapeutic drugs.

## Introduction

In 2021, lung cancer accounted for 25% of all cancer deaths on a global scale (1). Lung adenocarcinoma (LUAD) is one of the most predominant histological subtypes of lung cancer. Despite great advancements in the treatment of lung cancer, the 5-year survival rate for LUAD patients between 2010 and 2014 ranged from 10% to 20% in the majority of nations (2). Thus, identifying new biological markers and developing a comprehensive understanding of underlying treatment mechanisms for predicting effective therapies for LUAD are important.

Necroptosis, pyroptosis, and ferroptosis are types of regulated cell death (RCD) that have been discovered in addition to classical apoptosis. Ferroptosis, a form of RCD that is iron-dependent and triggered by an overabundance of lipid peroxides on cell membranes, is involved in the progression and treatment responsiveness in various malignancies (3). Notably, Tsvetkov and colleagues conceived a vital form of cell death termed cuproptosis (4). Excess intracellular copper induces the aggregation of lipoylated dihydrolipoamide S-acetyltransferase (DLAT), which is associated with the mitochondrial tricarboxylic acid (TCA) cycle and results in proteotoxic stress (4). It is important to note that the interconnection of a variety of cell death pathways occurs in many diseases ranging from intracellular infection to cancer. Furthermore, ferroptosis and necroptosis can be triggered by reactive oxygen species (ROS) and are both involved in ischemia–reperfusion-driven pathology. Strikingly, antitumor activity is produced when copper-transporting ATPase 1 (ATP7A) is degraded because this causes an increase in ROS as well as ferroptosis in colorectal cancer cells (5). However, the cross-talk between cuproptosis and ferroptosis and the therapeutic value of their interconnectivity is never explored.

The tumor microenvironment (TME), an important part of the tumor mass, which consists of tumor cells, immune cells, and stromal cells, has been reported to affect tumor prognosis and the tumor response for immunotherapy in LUAD (6–8). However, the immune mechanisms of TME in LUAD is not totally unclear. Thus, studies investigating the role of cuproptosis and its TME features are urgently needed.

In our study, we conducted a comprehensive pancancer genomic analysis by depicting the molecular correlations between cuproptosis and ferroptosis regulators in 33 cancer types, indicating the cross-talk between cuproptosis and ferroptosis regulators at the multiomic level. The CuFescore was established and validated to predict the response to immunotherapy and chemotherapeutic drugs. Thus, our study established an applied scoring scheme based on the regulators of cuproptosis/ferroptosis to identify LUAD patients eligible for immunotherapy and to predict sensitivity to chemotherapeutic drugs.

## Materials and methods

### Data acquisition

The Gene Expression Omnibus (GEO) and The Cancer Genome Atlas (TCGA) databases were accessed to acquire the LUAD RNA expression profile in addition to the accompanying comprehensive clinical annotations. **Supplementary Table S1** detailed the characteristics of 1274 samples belonging to 6 different cohorts (TCGA-LUAD, GSE30219 (9), GSE31210 (10), GSE3141 (11), GSE37735 (12), and GSE81089 (13)). Additionally, our research investigated two immunotherapy cohorts (GSE91061 (14) and GSE100797 (15)). The cuproptosis and ferroptosis regulators analyzed in our study are listed in **Supplementary Table S2**. FunRich 3.1.3 (accessed on 25 May 2022) was utilized to analyze the miRNAs’ targeted mRNAs, and the Kyoto Encyclopedia of Genes and Genomes (KEGG) was consulted for the enrichment of the miRNAs’ targeted signaling pathways. The Cancer 3’ UTR Atlas (TC3A, https://tc3a.org) (accessed on 6 July 2022) (16) provided the downloadable alternative polyadenylation (APA) sequence. Changes in the distal poly(A) site usage index (PDUI) may be utilized to quantify the different patterns of APA usage seen in each tumor and to identify 3’UTR shortening (negative index) and lengthening (positive index) (17).

### Single-cell RNA-sequencing analysis

ScRNA-seq data was extracted from GSE131907 (18) and processed by Cell Ranger (version 2.0.0, https://software.10xgenomics.com/single-cell/overview/welcome). Cell Ranger was utilized to measure the gene expression levels by processing the raw data from each sample. Following the completion of the quality check, cells that had between 200 and 10, 000 identified genes, and mitochondrial gene content of ≤ 20% were retained for subsequent analyses. Uniform manifold approximation and projections (UMAPs) were constructed utilizing the topmost 8 primary components. The predominant cell types were identified by the following markers: cancer cells (MDK, SOX4, EPCAM), alveolar cells (AGR3, FOLR1, SFTPD), epithelial cells (AGER, SFTPC, LAMP3, SCGB1A1, FOXJ1, RFX2), myeloid cells (C1QB, LYZ, CD68), endothelial cells (CLDN5, FCN3, RAMP2), fibroblasts (C1R, COL1A1, DCN), mast cells (CPA3, TPSAB1, TPSB2), B cells (CD79A, IGHG3, IGKC) and T cells (CD3D, TRAC, TRBC2).

### Genomic, transcriptomic, and clinical data analyzed across cancer types

The University of California, santa Cruz (UCSC) Xena browser (https://xena.ucsc.edu/) was accessed to extract the MC3 somatic mutation data and RNA sequencing data. In addition, we identified the interactions among cuproptosis and ferroptosis regulators based on the GeneMANIA interaction database (https://genemania.org) (19).

### Cell culture

Human NSCLC cell line A549 and bronchial epithelial cell line (BEAS-2B) were purchased from the American Type Culture Collection (ATCC, Manassas, VA, USA). Cells were maintained in RPMI-1640 medium containing 10% fetal bovine serum (FBS; Gibco, CA, USA) at 37 °C in a humidified atmosphere containing 5% CO_2_.

### RNA extraction and real-time PCR

Total RNA was extracted from cells with TRIzol reagent (Invitrogen, NY, USA). Chlorophorm isoamylalcohol was added and incubated for centrifugation. The aqueous phase was transferred and precipitated using isopropanol. The RNA pellet was washed with ethanol, air-dried and resuspended in RNase-free water. The concentration of RNA was measured by the spectrophotometer (NanoDrop, #ND-1000). RT-PCR assays were conducted to measure gene expression with a Prime Script RT reagent kit (TIANGEN, Beijing, China). The primers for the genes are listed in **Supplementary Table S3**. After demonstration that primer sets exert equal and high efciencies, relative expression was analyzed by 2−ΔΔCt method using the transcript levels of hypoxanthine–guanine phosphoribosyl transferase (HPRT) for normalization.

### Cell transfection

Cells were seeded in six well plates and the confluency was reached at 30% before the transfection. Non-specific scramble (scr) small-interfering RNA (siRNA) as a control and siRNA were transfected into cells with Lipofectamine 2000 (Invitrogen, CA, USA) according to manufacturer’s instructions. After transfection, cells were cultured for 48 h and treated as indicated. Cells were lysed for RNA isolation using TRIZOL method.

### Identification of cuproptosis/ferroptosis regulators based on the topology of the co-expression networks

To identify hub cuproptosis and ferroptosis regulators for each cancer type, we introduced the concept of “module” from the weighted gene coexpression network analysis (WGCNA) algorithm and treated the cuproptosis and ferroptosis regulators as a module (20). The overall expression level of the module was summarized as the module eigengene by the moduleEigengenes function in the R package WGCNA. We further calculated the module membership (i.e., module eigengene-based intramodular connectivity) as the link between the expression value of a given cuproptosis/ferroptosis regulator and the module eigengene. Hub cuproptosis/ferroptosis regulators were then defined as those that achieved a module membership greater than 0.4. The summary expression level of the identified hub cuproptosis/ferroptosis regulators was again calculated as epigenetic module eigengenes (EMEs) for each cancer type.

### Gene set enrichment analysis

Pathway studies were conducted for the purpose of assessing and comparing the 50 signature oncogenic pathways (21). The MSigDB database (h.all.v7.5.symbols.gmt) maintained by the Broad Institute served as the source for the acquisition of the signature gene set. After that, we assigned estimates of pathway activities to each sample by utilizing GSEA with the default parameters as defined in the clusterProfiler R package. This was done to keep the false discovery rate (FDR) under control.

### Unsupervised clustering for cuproptosis/ferroptosis regulators and principal component analysis

The “limma” R package was applied to normalize the data and detect genes with the prognostic values. The “drivers”, “markers”, “suppressors” from ferroptosis regulators and cuproptosis/ferroptosis regulators was calculated as four EMEs. To categorize LUAD patients into distinct subtypes depending on the findings of the research, an unsupervised clustering analysis was performed on the cuproptosis/ferroptosis regulators with the “ConsensusClusterPlus” package (22). The number of clusters (K) and their stability were determined by the consensus clustering algorithm and the R package “PCA” was conducted to verify the outcomes of the clustering.

### Gene set variation analysis

We applied the “GSVA” package in R to perform GSVA and examine the biological activities of the subtypes of cuproptosis/ferroptosis. The gene sets of “c2.cp.kegg.v7.5.symbols” were downloaded from MSigDB database for running GSVA analysis.

### Determination of differentially expressed genes between cuproptosis/ferroptosis subtypes

To determine the genes related to cuproptosis/ferroptosis regulators, we categorized the patients into three subtypes depending on the expression of the cuproptosis/ferroptosis-related genes. To discover DEGs between different subtypes, the empirical Bayesian approach of the “limma” R package was utilized.

### Establishment of CuFescore

We developed a scoring system to quantify the cuproptosis and ferroptosis regulators in each LUAD patient and the gene signature of cuproptosis and ferroptosis is termed CuFescore. The Cox regression model was used to reveal the genes with prognostic values. An unsupervised clustering analysis was utilized to detect overlapping DEGs and the prognostic DEGs were identified. To define the number of clusters and their stability, the consensus clustering algorithm was employed. The CuFescore was constructed by separating principal components 1 and 2. Collectively, we determined each patient’s CuFescore by applying a methodology that used in the prior research (23):

CuFescore = ∑(PC1_i_) + ∑(PC2_i_)

Where i indicates the expression of cuproptosis/ferroptosis-related genes.

### Mutation profiles

We extracted the mutation annotation format (MAF) from the TCGA database with the “maftools” R package to investigate the mutational landscape of LUAD patients between the high and low CuFescore groups. Co-occurrences were analyzed to determine the interaction of gene mutations.

### Prediction of the responsiveness to chemotherapy agents

To assess the different sensitivities to chemotherapeutic drugs between the low and high CuFescore groups, the pRRophetic algorithm was implemented in predicting the 50% inhibiting concentration (IC50) value of the 138 drugs (24).

### Statistical analysis

A Wilcox test was utilized for comparisons in the levels of RNA between tumor and non-tumor tissues. The time-dependent area under the receiver operating characteristic curve (AUC) was applied to evaluate the predictive power of CuFescore to survival of patients. The overall survival (OS) rates of each group were subjected to comparison via the use of a log-rank test in combination with a Kaplan-Meier analysis. Cox regression of OS was performed utilizing univariate data to find molecules linked to prognosis. Analysis of all statistical data was executed with R software (version: 4.0.5). Two-sided t-tests were employed for all of the statistical tests. Statistical significance was determined at *p* < 0.05.

## Results

### Genetic and transcriptional alterations of cuproptosis regulators in LUAD

**Figure 1:**
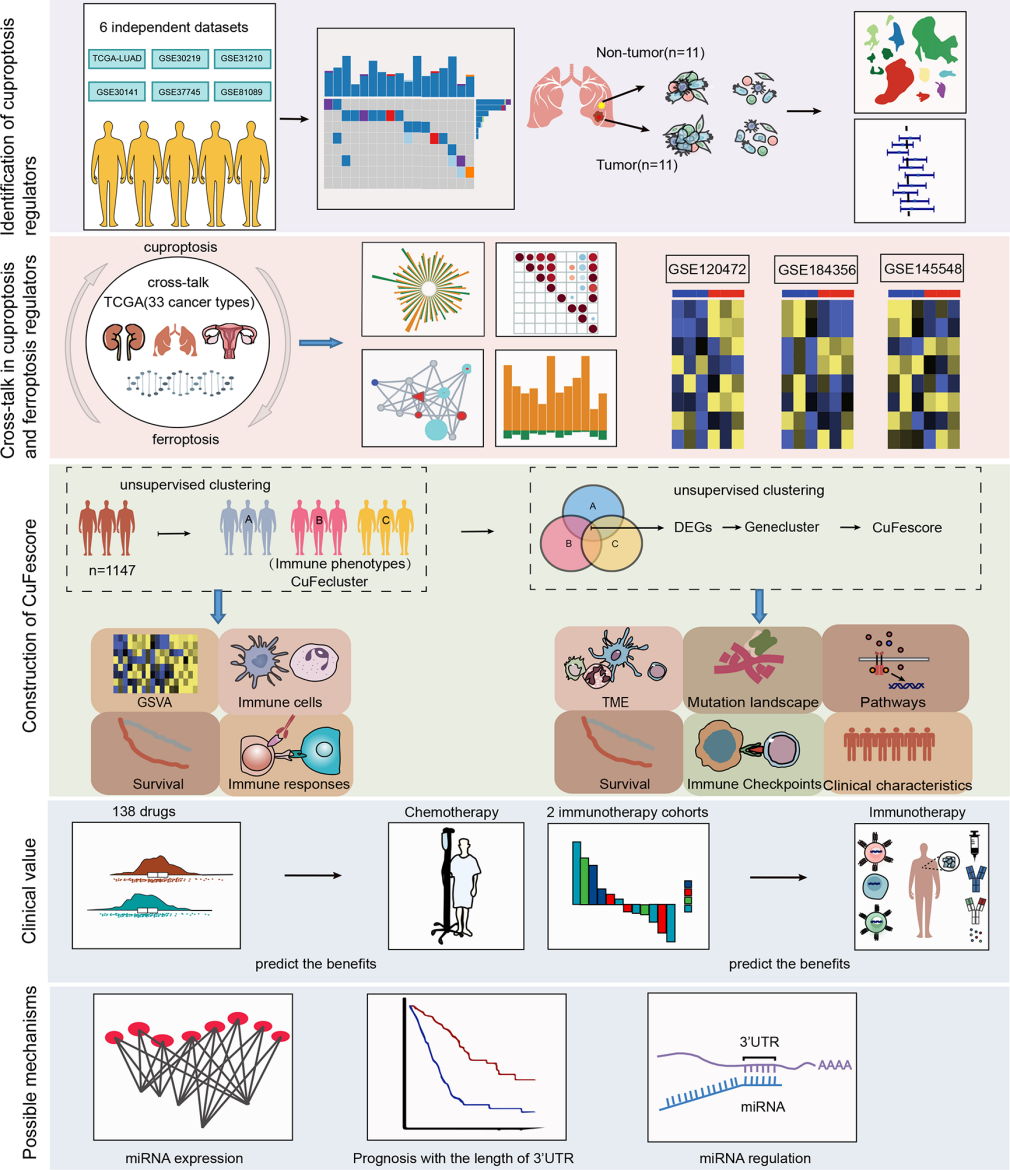
Workflow of our study.

**Figure 2:**
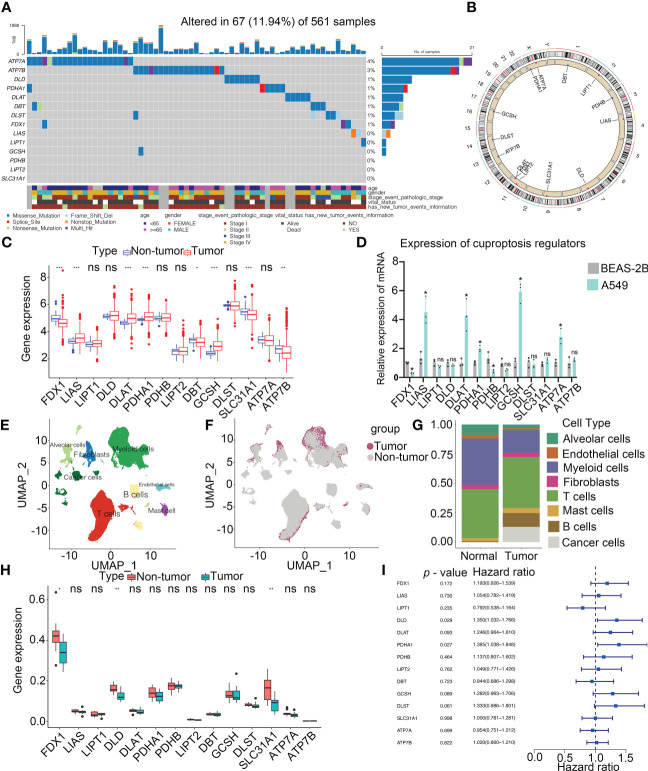
Landscape of genetic and expression variation of cuproptosis regulators in LUAD. **(A)** Mutation frequency of cuproptosis regulators in 561 LUAD patients from the TCGA cohort. Each column represents individual patients. The upper bar graph showed tumor mutational burden. The number on the right indicates the mutation frequency in the regulators. The right bar graph revealed the proportion of each variant type. The graph below determined clinical features of patients in the cohort. **(B)** Location of CNV alteration of cuproptosis regulators on 23 chromosomes. **(C)** Bulk sequencing showed the expression of cuproptosis regulators between adjacent non-tumor and LUAD samples in TCGA-LUAD cohort (n = 585). **(D)** Expression of cuproptosis regulators in NSCLC (A549) and normal lung epithelial cell line (BEAS-2B) by RT-PCR. **(E)** UMAP indicated the cell composition in the microenvironment of LUAD according to cell types. **(F)** Cell distribution originated from tumor and normal lung samples. **(G)** Bar plot determined the overall cell composition of normal and tumor samples. **(H)** ScRNA-seq analysis revealed the expression of cuproptosis regulators between adjacent non-tumor and LUAD samples in GSE131907 cohort (n = 22). **(I)** Forest plot showed the prognosis of cuproptosis regulators for LUAD patients in TCGA (n = 585). UMAP, uniform manifold approximation and projection. * *p* < 0.05, ** *p* < 0.01, *** *p* < 0.001, ns, not significant.

The workflow of this research is shown in **Figure 1**. Our study analyzed 14 cuproptosis regulators (4). To reveal the genetic alterations of cuproptosis regulators, we provided a brief overview of the incidence of non-silent somatic mutations in malignancies. In the TCGA cohorts of UCEC, BLCA, and CESC, the incidence of mutations of cuproptosis regulators was moderately high but was low in UVM (**Figure S1A**). Among 561 LUAD samples, 67 (11.94%) carried mutations of cuproptosis regulators (**Figure 2A**). The highest mutational frequency was observed in *ATP7A* (4%) and *ATP7B* (3%), while no mutations of *LIAS*, *LIPT1*, *GCSH*, *PDHB*, *LIPT2*, and *SLC31A1* were found. A significant mutation co-occurrence was exhibited between *GCSH*, *ATP7B*, *DBT*, *ATP7A*, and *DLST* (**Figure S1B**). However, there was no survival difference between patients with and without mutations in the TCGA-LUAD cohort (**Figure S1C**). The chromosomal locations of cuproptosis regulators were detected, as shown **Figure 2B**. In the TCGA-LUAD cohort, the mRNA expression of cuproptosis regulators was analyzed between adjacent non-tumor and LUAD samples (**Figure 2C**). The expression of cuproptosis regulators was analyzed in NSCLC (A549) and normal lung epithelial cells (BEAS-2B) (**Figure 2D**). Additionally, the exploration of CNV alteration frequency determined that there was a high incidence of CNV gains in the *LIPT2, SLC31A1*, and *DLD* (**Figure S1D**). To discover the relationship between the genetic variations and the mRNA expression, we found that *LIAS* with CNV gain shown high mRNA expression (**Figure 2C** and **S1D**). Interestingly, *DBT* and *FDX1* exhibiting a greater frequency of CNV loss revealed a high expression.

Single-cell profiling of tissues has emerged as an important tool for estimating the clinical relevance of different cell types in malignancies. After quality control, a whole-transcriptome database of 208506 cells from 11 LUAD and 11 non-tumor samples was analyzed. According to the cell-specific markers, we identified 8 cell types, including alveolar cells, B cells, cancer cells, fibroblasts, myeloid cells, T cells, endothelium cells, and mast cells (**Figure 2E** and **Figure S2A**). All the tumor cells were derived from the tumor samples (**Figures 2F, G** and **Figure S2B**). Notably, T cells accounted for the greatest percentage of all cell subsets in normal as well as cancerous tissue samples (**Figure 2G**). Based on the analysis of scRNA-seq, the expression of FDX1, DLD and SLC31A1 was much higher in non-tumor tissues than those in tumor tissues, consistent with our findings from a bulk sequencing analysis (**Figure 2H**). Meanwhile, the expression of cuproptosis regulators in each cell type was shown in **Figure S2C**. A univariate Cox regression analysis determined the prognostic significance of cuproptosis regulators in LUAD patients (**Figure 2I**). Low levels of 9 cuproptosis regulators were substantially associated with high OS rates in LUAD patients (**Figure S2D**). Thus, our results indicated the high heterogeneity of the genetic landscape and expression of cuproptosis regulators between non-tumor and LUAD samples, suggesting that the cuproptosis regulator expression imbalances were crucial in LUAD.

### Identification of novel interconnectivity between cuproptosis and ferroptosis regulators

**Figure 3:**
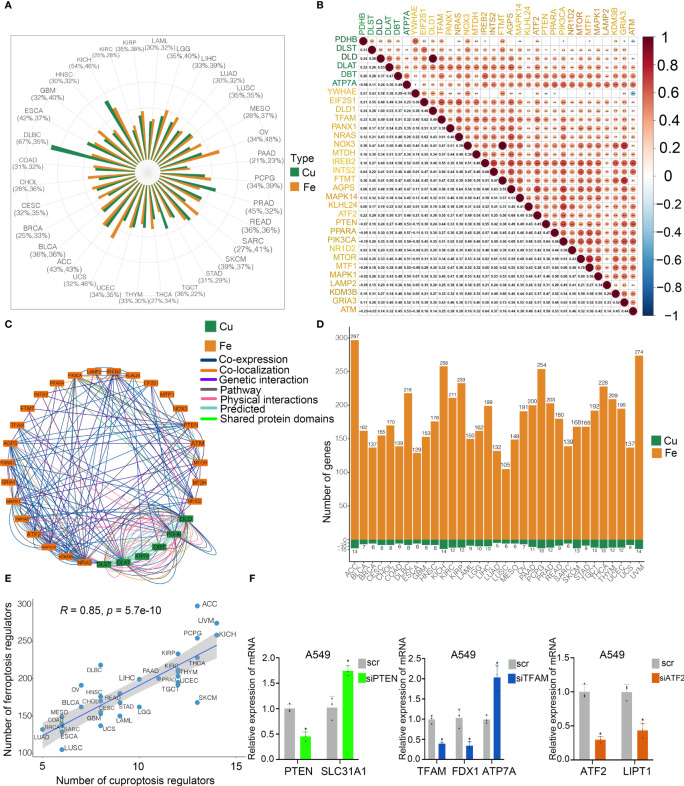
Cross-talk identified among cuproptosis and ferroptosis regulators in cancers. **(A)** Mutation frequency of cuproptosis and ferroptosis regulators in 33 cancer types. Green bar, mutation of cuproptosis regulators; Orange bar, mutation of ferroptosis regulators. **(B)** Co-occurrence of genetic alterations in the cuproptosis and ferroptosis regulators. Cuproptosis regulators are presented in green and ferroptosis regulators are in orange. **(C)** Protein-protein interactions among cuproptosis and ferroptosis regulators based on the GeneMANIA database. **(D)** Module membership-based hub cuproptosis and ferroptosis regulators across 33 cancer types. The lower panel shows the number of hub cuproptosis and ferroptosis regulators in each cancer type. **(E)** Correlations between the number of hub cuproptosis regulators and the number of hub ferroptosis regulators. The Pearson correlation coefficients (R) were analyzed for the correlation. **(F)** Levels of cuproptosis regulators after knockdown of ferroptosis regulators in A549 cells by RT-PCR. COAD, colon adenocarcinoma; DLBC, diffuse large B cell lymphoma; ESCA, esophageal carcinoma; GBM, glioblastoma; HNSC, head and neck squamous cell cancer; KICH, kidney chromophobe; KIRC, kidney renal clear carcinoma; KIRP, kidney renal papillary carcinoma; LAML, acute myeloid leukemia; LGG, low grade gliomas; LIHC, liver hepatocellular carcinoma; LUAD, lung adenocarcinoma; LUSC, lung squamous cell carcinoma; MESO, mesothelioma; OV, ovarian cancer; PAAD, pancreatic adenocarcinoma; PCPG, pheochromocytoma and paraganglioma; PRAD, prostate adenocarcinoma; READ, rectum adenocarcinoma; SARC, sarcoma; SKCM, skin cutaneous melanoma; STAD, stomach adenocarcinoma; TGCT, tenosynovial giant cell tumor; THCA, thyroid carcinoma; THYM, thymoma; UCEC, uterine corpus endometrial carcinoma; UCS, uterine carcinosarcoma; ACC, adenoid cystic carcinoma; BLCA, bladder carcinoma; BRCA, breast cancer; CESC, cervical squamous cell carcinoma and endocervical adenocarcinoma; CHOL; cholangiocarcinoma; UVM, uveal melanoma. * *p* < 0.05, ** *p* < 0.01, ns, not significant.

To explore the potential interconnectivity in various cell death pathways, we investigated the cross-talk between the cuproptosis and ferroptosis regulators. Genomewide omics data for 33 cancer types from TCGA were obtained for analysis. We found that most of cuproptosis and ferroptosis regulators exhibited comparable frequencies of mutations across 33 cancer types (**Figure 3A**). Moreover, our findings indicated strong correlations between cuproptosis and ferroptosis regulators (**Figure 3B**). Cuproptosis regulators interacted with ferroptosis regulators from the GeneMANIA database (**Figure 3C**). To determine the hub regulators involved in the interconnectivity, we performed WGCNA to identify hub genes in the cuproptosis and ferroptosis regulators among 33 cancer types (**Figure 3D**). Interestingly, the number of hub cuproptosis regulators was strongly associated with that of hub ferroptosis regulators in distinct cancers (R = 0.86; **Figure 3E**), indicating the possible cross-talk between cuproptosis and ferroptosis regulators in distinct cancers. We also investigated the activity of hallmark oncogenic pathways in various cancers (**Figure S3**). To validate the regulation between cuproptosis and ferroptosis regulators, we analyzed the expression of cuproptosis regulators after the knockdown of several ferroptosis regulators in the previous studies (**Figure S2E**). In the GSE120472 cohort, knockout of Pten in primary mouse embryonic fibroblasts (MEFs) resulted in the upregulation of 3 cuproptosis regulators including Dbt, Slc31a1 and Atp7a. In the GSE184356 cohort, knockdown of TFAM led to a significant change in PDHA1, PDHB, ATP7A and ATP7B in human dermal fibroblasts. In the GSE145548 cohort, knockdown of ATF2 in breast cancer cells MCF7 resulted in the dramatic change of cuproptosis regulators (DLST, GCSH, PDHA1, LIPT1 and DLD). Moreover, we transfected siRNAs and shRNA into A549 cells to validate the correlation between cuproptosis and ferroptosis regulators. The expression of SL31A1 was upregulated after the knockdown of PTEN, while the levels of ATP7A were increased after the knockdown of TFAM and LIPT1 was inhibited after the knockdown of ATF2 (**Figure 3F**), suggesting a strong association between cuproptosis and ferroptosis regulators. Thus, our results indicated the cross-talk and biological regulation between cuproptosis and ferroptosis regulators in cancers.

### TME cell infiltration characteristics in distinct patterns of cuproptosis and ferroptosis regulators

**Figure 4:**
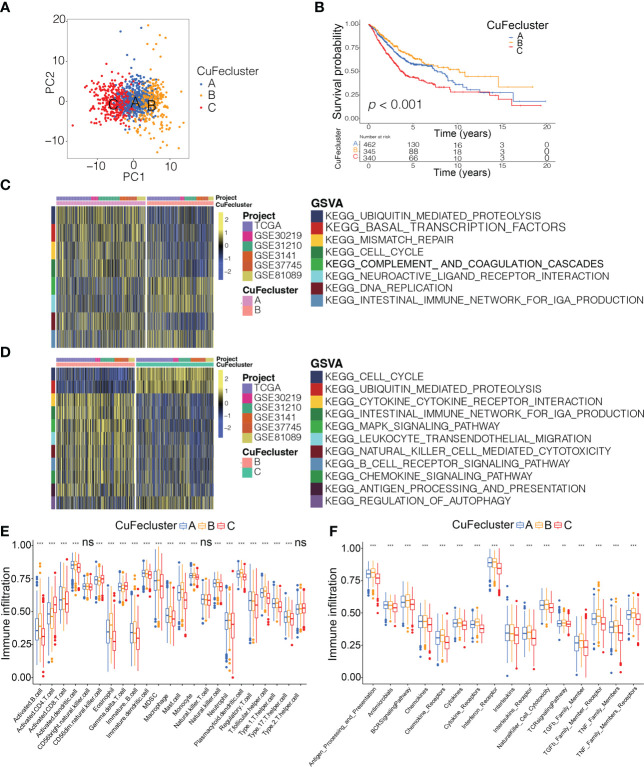
Tumor microenvironment cell infiltration characteristics and transcriptome traits in distinct CuFeclusters. **(A)** Principal component (PC) analysis revealed remarkable difference between three CuFeclusters from 6 cohorts (n = 1147). **(B)** Kaplan-Meier curves of survival for three patterns of three CuFeclusters based on LUAD patients from six cohorts (TCGA-LUAD, GSE30219, GSE31210, GSE3141, GSE37745, and GSE81089). **(C, D)** GSVA enrichment analysis shown the activation states of biological pathways in distinct CuFeclusters. The heatmap was used to visualize these biological processes, and yellow represented activated pathways and blue represented inhibited pathways. **(C)** CuFecluster A vs **(B, D)** CuFecluster B vs **(C, E)** Characteristics of immune infiltrating cells in different CuFeclusters. **(F)** Characteristics of immune responses in different CuFeclusters. GSVA, gene set variation analysis. ** *p* < 0.01, *** *p* < 0.001, ns, not significant.

By conducting unsupervised clustering based on the levels of cuproptosis and ferroptosis regulators, the patients from 6 cohorts (TCGA, GSE30219, GSE31210, GSE3141, GSE37745 and GSE81089; n = 1147) were divided into three subtypes, named CuFecluster A/B/C (**Figure S4A, B**). PCA illustrated a relatively evident distinction existed in the 3 clusters (**Figure 4A**). Patients in the CuFecluster B had a more favorable prognosis compared to ones in CuFecluster A and C (*p* < 0.001, **Figure 4B**). GSVA enrichment pathways were carried out in 1147 patients from 6 different cohorts to determine the biological functions of 3 CuFeclusters. Compared with CuFecluster A and C, CuFecluster B was associated with immune fully activation including B cell receptor signaling pathway, natural killer cell mediated cytotoxicity, antigen processing and presentation, cytokine-cytokine receptor interaction and chemokine signaling pathway (**Figures 4C, D**). Moreover, CuFecluster B was rich in the infiltration of various activated immune cells (**Figure 4E**). Considering a corresponding survival advantage, CuFecluster B was categorized as an immune-inflamed phenotype. This phenotype is distinguished by the presence of adaptive immune cell infiltration as well as immune activation. CuFecluster A was associated with several cell proliferation processes notably, mismatch repair, DNA replication, and cell cycle (**Figure 4C**), while CuFecluster A was relatively highly correlated with the innate immune cells including MDSC, eosinophil, natural killer, monocyte, mast cell, and macrophage (**Figure 4E**). Interestingly, CuFecluster A was also highly associated with TGF-β family member and TGF-β family member receptor (**Figure 4F**). A previous research has suggested that the immune-excluded phenotype is distinguished by the presence of a large number of immune cells and an elevated level of activity in the TGF-β signaling pathway, whereas immune cells were unable to penetrate the parenchyma of the tumors because they were hampered in the stroma that was enclosing the nests of tumor cells. Therefore, it was determined that CuFecluster A represented the immune-excluded subtype. In addition, CuFecluster C was found to obtain a low number of immune cells and a suppressed immunological response (**Figures 4D–F**), accordant with the main characteristics of the immune-desert phenotype. Thus, there was a remarkable difference between the three CuFeclusters in terms of the cell infiltration characteristics of the TME.

### Identification of cuproptosis/ferroptosis regulators-related gene subtypes and establishment of CuFescore

**Figure 5:**
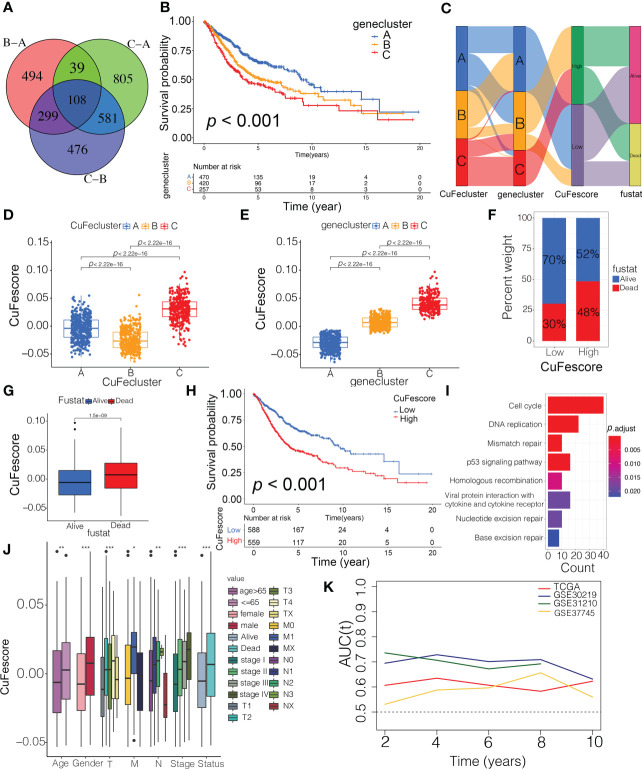
Construction of the CuFescore and the prognostic values of the CuFescore. **(A)** Overlapped cuproptosis/ferroptosis-related genes shown in Venn diagram. **(B)** Kaplan-Meier curves of survival in 6 cohorts with three distinct geneclusters. **(C)** Alluvial diagram showing the changes in CuFeclusters, geneclusters and CuFescores. CuFescore in distinct **(D)** CuFeclusters and **(E)** geneclusters. **(F)** Proportion of survival and death in the high and low CuFescore groups. **(G)** Comparison of the CuFescore in alive versus dead patients. **(H)** Kaplan-Meier curves of survival in the high and low CuFescore groups. **(I)** Functional annotation for DEGs between the low and high low CuFescore groups using GO enrichment analysis. The color depth of the barplots represented the number of genes enriched. **(J)** Difference in CuFescore among distinct clinical subgroups in LUAD cohort. **(K)** Time-dependent AUC value in TCGA-LUAD, GSE30219, GSE31210 and GSE37745. AUC, area under curve. * p < 0.05, ** *p* < 0.01, *** *p* < 0.001.

To evaluate the possible genetic modifications depending on the distinct cuproptosis/ferroptosis subgroups, we got 108 overlapped DEGs (**Figure 5A**) and found 105 DEGs with the prognostic significance by a univariate Cox regression analysis (**Supplementary Table S4**). We carried out an unsupervised cluster analysis and categorized the patients into 3 unique genomic subtypes, which we referred to as genecluster A/B/C (**Figures S4C and D**). Notably, a significantly improved prognosis was found in genecluster A compared to the other clusters (*p* < 0.001, **Figure 5B**). Even though our research showed a cuproptosis/ferroptosis-associated gene alteration in the prognosis, we generated applied scores for predicting cuproptosis and ferroptosis modification in individual patients as per the expression of the cuproptosis/ferroptosis-related DEGs. An alluvial diagram illustrates the steps involved in the establishment process of CuFescore (**Figure 5C**). We found on the evaluation that patients in CuFecluster B (**Figure 5D**) and genecluster A (**Figure 5E**) had low CuFescores. Additionally, we examined the overlap of the 3 distinct subtypes. CuFecluster A accounted for 36.6% of the patients in the high CuFescore group, and in the low CuFescore group, 52.4% of samples overlapped with CuFecluster B (**Figure S5A**). Meanwhile, in the high CuFescore group, 55% of cases overlapped with genecluster B, whereas in the low CuFescore group, 82% of cases overlapped with genecluster A (**Figure S5B**). When compared to the survival rate of the cohort with a high CuFescore, the subgroup with a low CuFescore had a much better probability of surviving (70% vs 52%, **Figure 5F**), comparable to the findings in early- (T1-2) (**Figure S5C**) and advanced- (T3-4) stage of lung cancer (**Figure S5D**). Consistent with this finding, the mean CuFescores were much lower in alive cases compared to those in the dead cases (**Figure 5G**). The Kaplan-Meier analysis showed a better prognosis for patients in the low CuFescore group (*p* < 0.001, **Figure 5H**). The stability of the CuFescore model was validated in 4 independent LUAD cohorts to validate the prognostic values (**Figure S5E-H**). Enriched pathways in the low CuFescore group were DNA replication, mismatch repair and cell cycle (**Figure 5I**). In addition, it was shown that patients with low CuFescores had a correlation with early clinical and pathological characteristics and stages (**Figure 5J**), which revealed that these individuals had a survival advantage characterized by the CuFecluster B and immune-inflamed subtype. In addition, time-dependent AUC curves determined that the CuFescore functioned as a predictive biological marker for the OS of LUAD patients in the 4 cohorts (**Figure 5K**). Therefore, these data showed that the CuFescore was associated with LUAD patients’ prognoses.

### Association between the CuFescore and immune checkpoints

**Figure 6:**
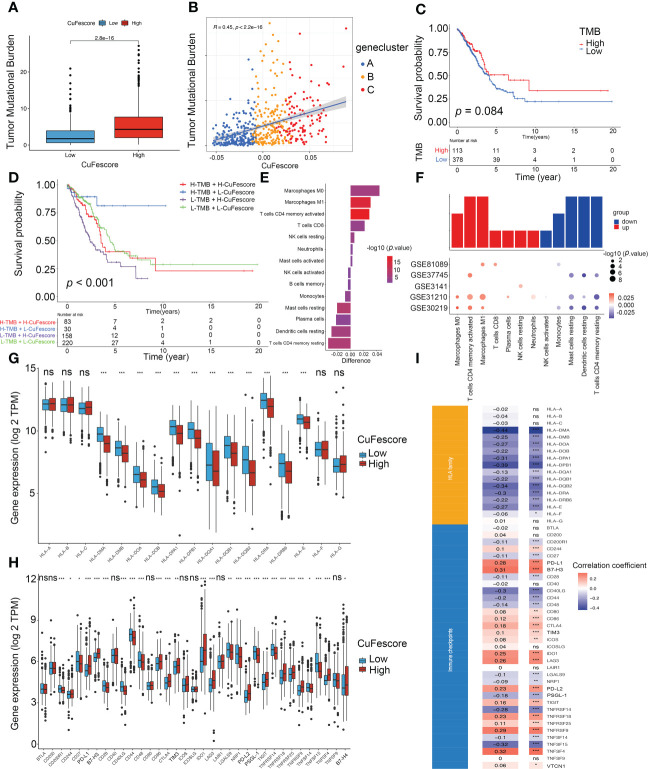
Correlation between the CuFescore and immune checkpoints. **(A)** Comparison of TMB in the high and low CuFescore group. **(B)** Correlation between CuFescore and TMB. **(C)** Kaplan-Meier curves of survival in the high and low TMB groups. **(D)** Survival analyses for patients stratified by both CuFescore and TMB using Kaplan-Meier curves. **(E)** Difference in the relative abundance of immune cell infiltration in tumor microenvironment between the high and low CuFescore groups. Difference > 0 indicates that the immune cells were enriched in the low CuFescore group, and the column color represents the statistical significance of the difference. **(F)** Expression of cell types in the five cohorts. Analyses for the expression of **(G)** HLA family genes and **(H)** immune checkpoints in the CuFescore groups. **(I)** Correlation analysis for CuFescore and the expression of HLA family genes and immune checkpoints. TMB, tumor mutational burden; TPM, transcript per million. * *p* < 0.05, ** *p* < 0.01, *** *p* < 0.001, ns, not significant.

Based on the strong correlation with immune-related pathways including immune checkpoints, CD8 T effector and antigen processing machinery (**Figure S5I**), we hypothesized that the CuFescore is associated with immunotherapy. In this study, we examined immunotherapy-related parameters such as tumor mutational burden (TMB) and immunological checkpoints. Higher TMB was found in the high CuFescore group in contrast with the low CuFescore group (*p* = 2.8e-16; **Figure 6A**). Moreover, the CuFescore was also positively correlated with TMB (R = 0.45, *p* < 2.2e-16, **Figure 6B**). No difference was found between the low and high TMB subgroups (*p* = 0.084, **Figure 6C**). By combining the CuFescore and TMB, we noted that patients with a low CuFescore and high TMB patients exhibited a favorable prognosis (*p* < 0.001, **Figure 6D**). The CuFescore was associated with tumor-infiltrating immune cells (TIICs), comprising activated dendritic cells, activated CD4 T and CD8 T cells, as well as activated B cells (**Figure S5J**). We also evaluated the differences in TME cells between the two CuFescore groups. The findings illustrated that the low CuFescore exhibited an elevated infiltration level by M0 and M1 macrophages, T cells CD4 memory activated, T cells CD8, NK cells resting, Neutrophils, and mast cells activated, whereas the high CuFescores had elevated levels of macrophages, activated CD4 T cells, and activated mast cells (**Figures 6E, F**), demonstrating that the patients with low CuFescores were immune activation. Overall, our findings presented proof that the CuFescore was related to the immune signature including TMB and infiltrating immune cells.

The Wilcoxon test indicated that there were substantial variations between the 2 CuFescore groups in terms of the expression of 12 HLA family genes (**Figure 6G**) and 27 immune checkpoints (**Figure 6H**). In addition, the CuFescore was significantly correlated with 13 HLA family genes and 29 immune checkpoint expression (**Figure 6I**). Therefore, the data showed the CuFescore was highly associated with tumor immune checkpoints.

### Mutation status in the high and low CuFescore groups

**Figure 7:**
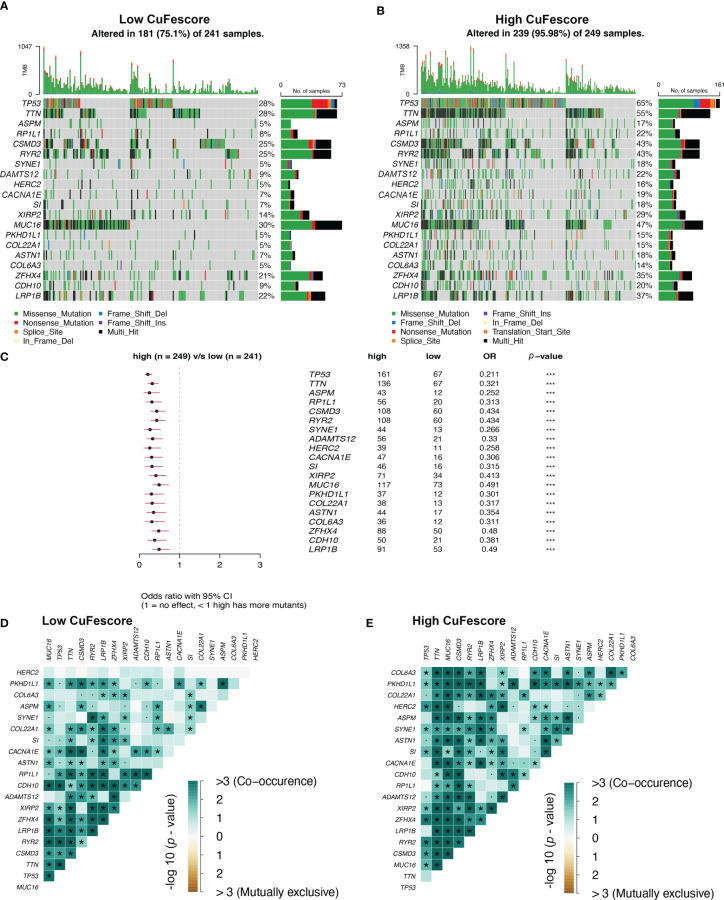
Association between the CuFescore and tumor mutation status. Visual summary showing common genetic alterations in the **(A)** low and **(B)** high CuFescore groups. **(C)** Forest plot of gene mutations in the patients. Interaction effect of genes mutating differentially in patients in the **(D)** low and **(E)** high CuFescore groups. *p* < 0.1, * *p* < 0.05, *** *p* < 0.001.

To additionally examine the correlation between the CuFescore and mutations in LUAD, we determined somatic mutations from TCGA cohort between high and low CuFescore groups. The genes frequently mutated are displayed in **Figures 7A, B**. Remarkably, mutations in 20 genes were found to be more frequent in patients with high CuFescores (**Figure 7C**). Additionally, significant co-occurrences were discovered between mutations of these genes in both the low (**Figure 7D**) and high CuFescore groups (**Figure 7E**).

### The CuFescore predicted chemotherapeutic and immunotherapeutic benefits

**Figure 8:**
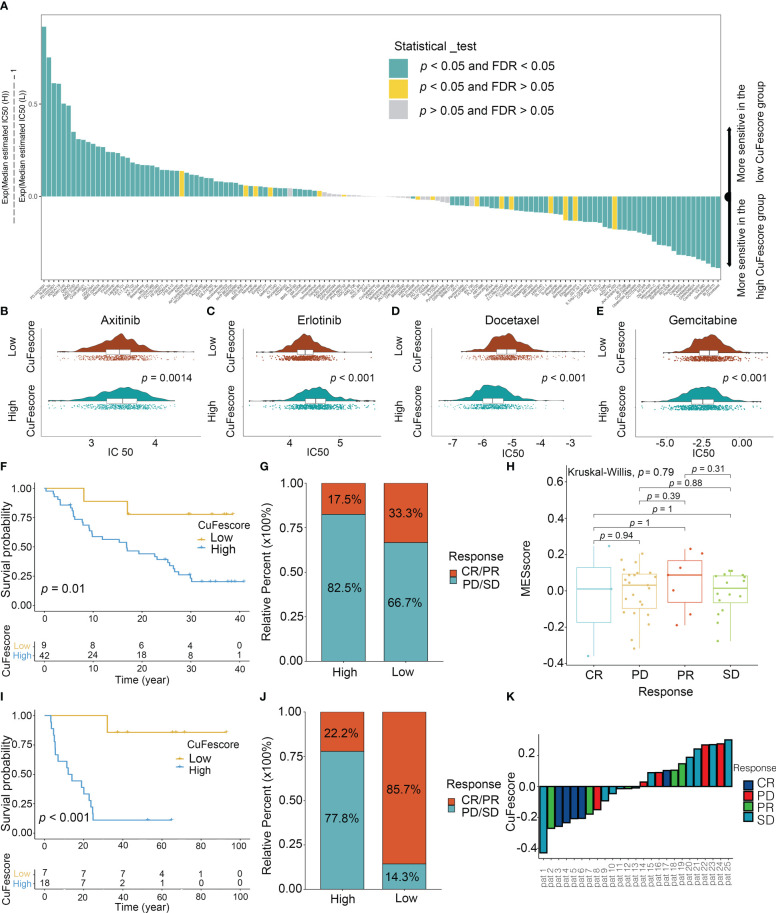
Role of the CuFescore in the chemotherapy and immunotherapy. **(A)** Sensitivity of 138 drugs. Efficacy of **(B)** axitinib; **(C)** erlotinib; **(D)** docetaxel and **(E)** gemcitabine. **(F)** Kaplan-Meier curves of survival in the patients receiving anti-PD-L1 therapy in GSE91061. **(G)** Proportion of patients with response to PD-1 blockade immunotherapy in the high and low CuFescore groups. **(H)** Distribution of CuFescore in distinct anti-PD1 clinical response groups. **(I)** Kaplan-Meier curves of survival i8n the patients receiving adoptive T cell therapy in GSE10797. **(J)** Proportion of patients with response to adoptive T cell therapy in the high and low CuFescore groups. **(K)** Correlation of CuFescore with clinical response to adoptive T cell therapy. IC 50, half maximal inhibitory concentration; FDR, false discovery rate; MEscore, module eigengene score; CR, complete response; PD, progressive disease; PR, partial response; SD, stable disease.

To assess the value of the CuFescore for predicting the responsiveness to chemotherapy drugs, the IC50 values of 138 drugs were calculated (**Figure 8A**, **Supplementary Table S5**). Patients with low CuFescores had strong sensitivity to axitinib (*p* = 0.0014, **Figure 8B**) and erlotinib (*p* < 0.001, **Figure 8C**) while those in the high-CuFescore group exhibited strong sensitivity to docetaxel (*p* < 0.001, **Figure 8D**) and gemcitabine (*p* < 0.001, **Figure 8E**), indicating that the CuFescore might be used as a predictive biological marker for medications against LUAD.

To explore the predictive values of the CuFescore regarding the response to immune checkpoint blockade (ICB) treatment, we analyzed 2 immunotherapy cohorts (GSE91061 and GSE100797) with the CuFescore. We calculated the CuFescore in the patients who received the immunotherapy depending on the levels of cuproptosis/ferroptosis regulators-related genes and categorized them into high and low CuFescore groups. In the GSE91061 cohort, patients with low CuFescores had a better prognosis in contrast to those in the high CuFescore group with anti-CTLA4 and anti-PD1 treatment (*p* = 0.01, **Figure 8F**). Patients with low CuFescores exhibited remarkable therapeutic benefits and improved immune sensitivity to the PD-1 blockade (responser/nonresponser: 33.3%/17.5%, **Figure 8G**), even though there was no CuFescore difference between complete response (CR), progressive disease (PD), partial response (PR) and stable disease (SD) patients (**Figure 8H**). Moreover, this finding was also validated in the GSE100797. Patients with low CuFescores had a prolonged survival (**Figure 8I**) and significantly better therapeutic outcomes (responser/nonresponser: 85.7%/22.2%, **Figure 8J**). The significant clinical response to adoptive T cell therapy in patients with low CuFescores in contrast with those with high CuFescores was verified (**Figure 8K**). Collectively, the CuFescore had a substantial correlation with tumor immune phenotypes and was effective in predicting the responsiveness of patients to ICB therapy.

### The CuFescore was correlated with miRNA and post-transcriptional regulation

**Figure 9:**
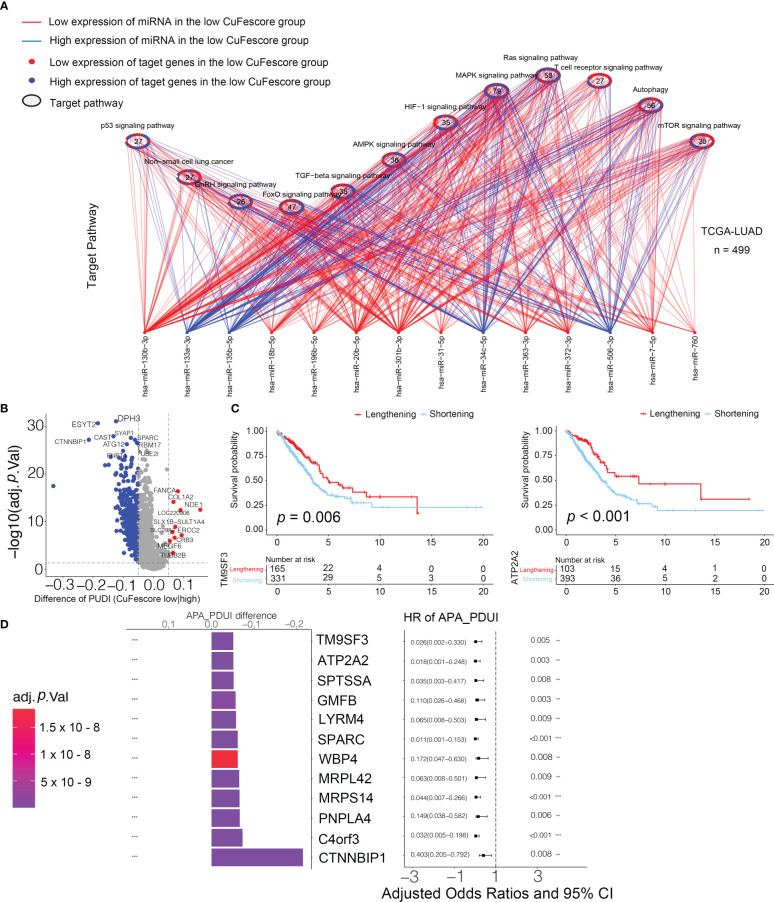
Associated between the CuFescore and the post-transcriptional characteristics. **(A)** Differences in miRNA-targeted signaling pathways in the TCGA-LUAD cohort between the high and low CuFescore groups. **(B)** Differences in the distal poly **(A)** site usage index (PDUI) of each gene between the high and low CuFescore groups. Red, PDUI lengthening; blue, PDUI shortening; Grey, no significant change in PDUI. **(C)** Kaplan-Meier curves indicated overall survival between PDUI lengthening (red) and PDUI shortening (blue) of TM9SF3 and ATP2A2. **(D)** Bar graphs showed the difference in the distal poly **(A)** site usage index (PDUI), and the forest plots showed univariate Cox regression analyses for PDUI differential genes between the high and low CuFescore groups. APA, alternative polyadenylation. HR, hazard ratio. CI, confidence interval. ** *p* < 0.01, *** *p* < 0.001.

The CuFescore is an assessment system depending on the cuproptosis and ferroptosis regulators, which are found in the association of post-transcriptional modifications. To evaluate the regulation of the CuFescore in the interpretation of transcriptional and post-transcriptional events, we analyzed APA events. Given that transcripts processed by APA have a short 3’UTR, thus tolerating the regulation of miRNAs, we hypothesized that the CuFescore is strongly associated with the expression miRNAs as potential mechanisms under the action of APA events. In the TCGA-LUAD cohort, we detected 79 differentially expressed miRNA between high and low CuFescore groups. There was an enrichment of miRNA-targeted genes involved in the autophagy, ROS signaling pathway, MAPK signaling pathway, and other pathways (**Figure 9A**). The expression levels of 29 out of 56 miRNA-targeted genes involved in autophagy were found to be elevated. Additionally, the cGMP-PKG signaling pathway (11/22) and the cAMP signaling pathway (11/23) were enriched among the miRNAs targeted genes that had lowered expression levels in the high CuFescore group. As per the obtained findings, the CuFescore had a very strong link to the expression of miRNA as well as the modulation of signaling pathways.

We examined the APA events to discover the connection between the CuFescore and the post-transcriptional features. We found the genes with the APA differences between high and low CuFescore groups and examined the prognostic significance to show whether the survival of LUAD patients is affected by the length of 3’UTR (**Figure 9B**). Genes with lengthening APA events were in the low CuFescore group, consistent with prolonged survival (**Figure 9C**). TM9SF3 and ATP2A2 were considered as proto-oncogenes in leukemia (25), triple-negative breast cancer (26) and diffuse astrocytic tumor (27). There was a correlation between the short transcripts of 2 genes and the worse prognosis of individuals (**Figure 9D**). Additionally, TM9SF3 was targeted directly by miR-1193 on 3’UTR (25). We were under the impression that since the 3’-UTR of genes had been shortened, miRNA may not bind to the genes, thereby removing the inhibitory effects on proto-oncogenes and enhancing the advancement of LUAD.

## Discussion

A recent study illustrated that intracellular copper (Cu) generates a unique type of RCD that is distinct from oxidative stress-associated cell death, termed cuproptosis. Some research advances have highlighted the importance of cuproptosis in the progression of clear cell renal cell carcinoma (ccRCC) (28) and hepatocellular carcinoma (HCC) (29). To further understand the integrated roles of cuproptosis regulators, we explored global alterations in cuproptosis regulators at the genetic and transcriptional levels and their mutual association in LUAD. One pioneering study reported that the flexible usage and interconnectivity of diverse cell death pathways protect against intracellular infection (30). Our study was the first one to focus on the interconnectivity between cuproptosis and ferroptosis in the pancancer analysis using a multiomics approach. The comparable frequencies between cuproptosis and ferroptosis regulators provided evidence of interconnectivity. The strong expression correlation between some cuproptosis and ferroptosis regulators supports this finding. Moreover, among 33 cancers the number of cuproptosis regulators was highly associated with that of ferroptosis regulators. Meanwhile, it provided the evidence that the strong interaction between cuproptosis and ferroptosis regulators existed not only in LUAD but also in other cancers. Notably, after the knockout/knockdown of several ferroptosis regulators, cuproptosis regulators were significantly regulated, indicating their biological interconnectivity. Interestingly, exploring the potential interconnectivity between cuproptosis and ferroptosis will offer deeper insights into the TME antitumor immune response, and guide the establishment of effective immunotherapeutic strategies. It was reported that the combined application of the copper chelator elesclomol and copper leads to copper blocking in mitochondria due to the loss of the cuproptosis regulator ATP7A, further enhancing oxidative stress and consequent ferroptosis in colorectal cancer cells (5). The mechanisms of the interconnectivity deserve further analysis.

We then identified 3 distinct cuproptosis/ferroptosis regulator clusters, named CuFecluster A/B/C. The three CuFeclusters presented significantly different TME cell infiltration characteristics. CuFecluster B was associated with immune activation and a better prognosis and was considered an immune-inflamed phenotype. CuFecluster A was distinguished from other clusters by having a large number of innate immune cells as well as activation of the TGF-β signaling pathway, both of which correlate with an immune-excluded subtype. Rather than penetrating the parenchyma of these tumors, the immunocytes remain in the stroma surrounding the nests of tumor cells, and as a result, the patient’s survival does not improve. Immune suppression was observed in CuFecluster C, which is consistent with an immune-desert subtype. Therefore, the features of TME cell infiltration found in the three different CuFeclusters were extremely congruent with 3 different immune phenotypes.

Patients were classified into 3 geneclusters to explore the possible genetic modifications associated with the different CuFeclusters. CuFescore is a comprehensive and robust scoring system that takes into account the heterogeneity and complexity of individuals. It was utilized to quantify the cuproptosis/ferroptosis-related patterns of each patient based on the expression of DEGs. Importantly, patients with low CuFescores were found to have a favorable prognosis. Furthermore, the significantly prolonged OS of the patients with low CuFescores and high TMB enhanced the advantage of low CuFescores. TMB and the expression of immune checkpoints are well recognized to influence immunotherapy effectiveness. Several key members of the HLA family and critical genes, such as B7-H3, TIM3, PD-L1, and B7-H4, were differentially expressed in the low and high CuFescore groups. In addition, a remarkable correlation was validated between the CuFescore and immune checkpoints. Collectively, the findings suggested that the CuFescore plays a role in immunotherapy for LUAD patients.

Mutation is an unavoidable factor in the therapeutic effect of immunotherapy. Patients with low CuFescores exhibited a longer prognosis and contained more mutations in 15 novel genes. Previous studies reported that TP53 mutations decrease the antitumor immune response as well as the responsiveness of tumors to immunotherapy, similar to our findings. In addition, PD-1 inhibitors revealed significant therapeutic benefits when combined with co-occurring mutations in patients. Fewer co-mutations occurred in the low CuFescore group with the positive effect of immunotherapy, consistent with our previous results.

To explore the predictive values of the CuFescore for chemotherapy and immunotherapy, we found that patients with low CuFescores were more sensitive to axitinib and erlotinib, while patients with high CuFescores were more sensitive to docetaxel and gemcitabine. In recent years, immunotherapy has emerged as a promising new therapeutic option for a variety of malignancies, particularly LUAD. To test our hypothesis that the CuFescore is a reliable scoring system to assess LUAD patient eligibility for immunotherapy, we applied the CuFescore in two independent immunotherapy cohorts. Within the cohorts, a favorable prognosis of LUAD patients was correlated with high CuFescores. Patients with low CuFescores were shown to benefit more from PD-L1 inhibition, both in terms of its therapeutic effects and immune responses. A combination of the results from the two immunotherapy cohorts highly supported the supposition that the CuFescore is a predictor of LUAD patient immunotherapeutic response. Overall, we consider the CuFescore as a predictor for evaluating drug sensitivity and clinical responsiveness to immunotherapy in LUAD patients. To explore the possible mechanism of CuFescore, we discovered that the CuFescore was associated with the expression of miRNA and that miRNA might target the 3’UTR of genes, regulating gene expression and participating in cancer progression.

However, several limitations should be considered in our study. First, our study was mainly based on integrative bioinformatics, and a selection bias is inherent to the design. Second, even though some key findings were supported by experimental validation, further experiments are required to explore the potential mechanisms including the interaction between cuproptosis and ferroptosis. Finally, the patients in this study were from two immunotherapeutic cohorts (GSE91061 and GSE10797); GSE91061 focused on the patients with advanced melanoma while GSE10797 focused on the patients diagnosed with breast cancer. Clinical studies with LUAD patients are needed to verify our findings in the immunotherapy.

## Conclusion

In conclusion, we established a CuFescore model to predict the prognosis of LUAD patients, which was strongly correlated with immune checkpoints and mutations. The CuFescore is an applied scoring system for evaluating the sensitivity to chemotherapeutic drugs and identifying LUAD patients eligible for immunotherapy.

## Data availability statement

The original contributions presented in the study are included in the article/**Supplementary Material**. Further inquiries can be directed to the corresponding authors.

## Author contributions

YS, YP and HL supervised the project and designed this study. YS, DL, MY and QL organized the public data and prepared all the figures and tables. YS and MY conducted the data analysis. YS, DL and QL drafted the manuscript. QL, YP and HL revised the manuscript. All authors listed have made a substantial, direct, and intellectual contribution to the work and approved it for publication.
