# Supplementary figures and images for: Cross-talk between cuproptosis and ferroptosis regulators defines the tumor microenvironment for the prediction of prognosis and therapies in lung adenocarcinoma
Source: Front Immunol. 2023 Jan 17;13:1029092. doi: 10.3389/fimmu.2022.1029092 (PMC9887127; doi:10.3389/fimmu.2022.1029092)

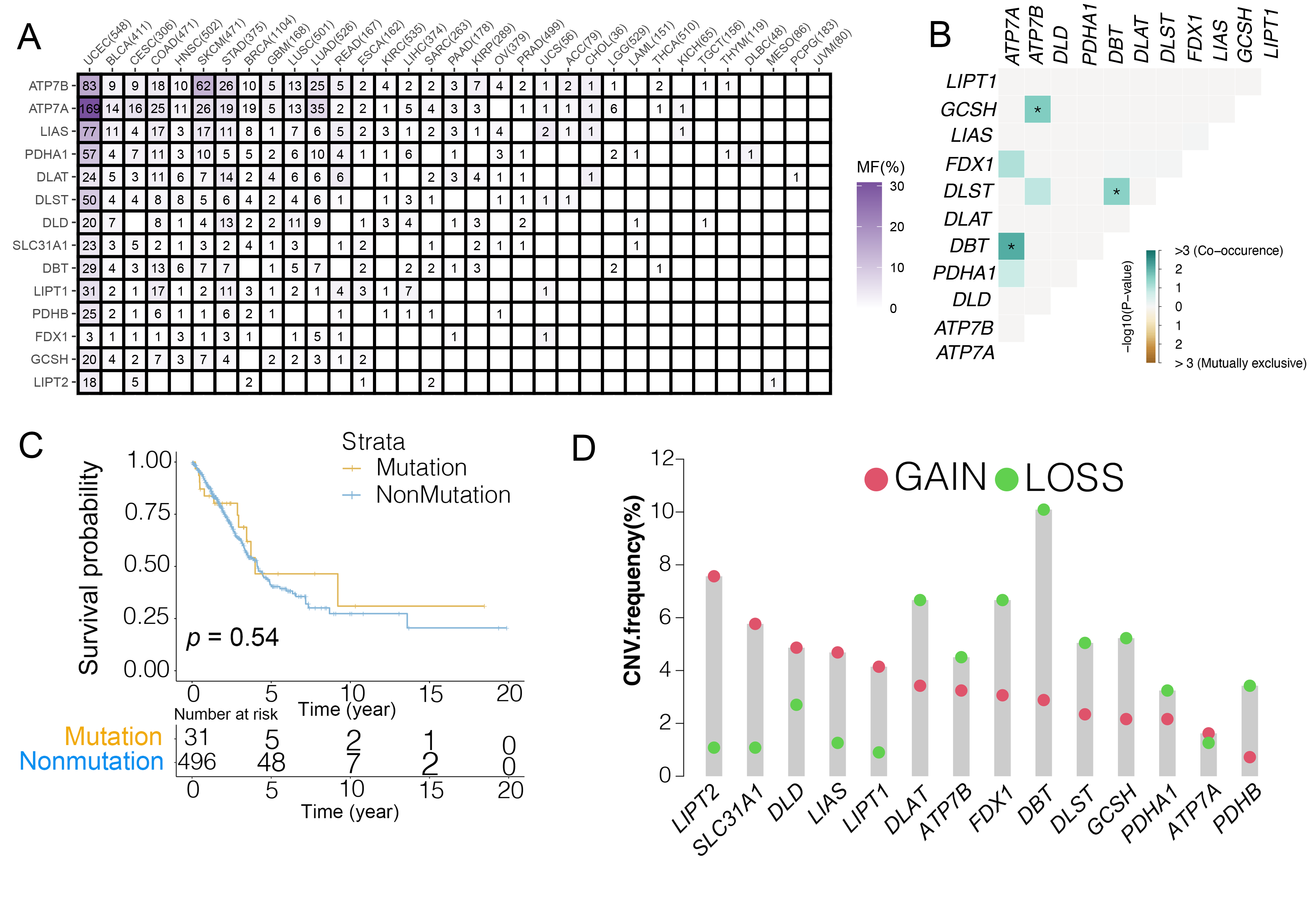

Supplement: Supplementary file 2 [file Image_1.tif]

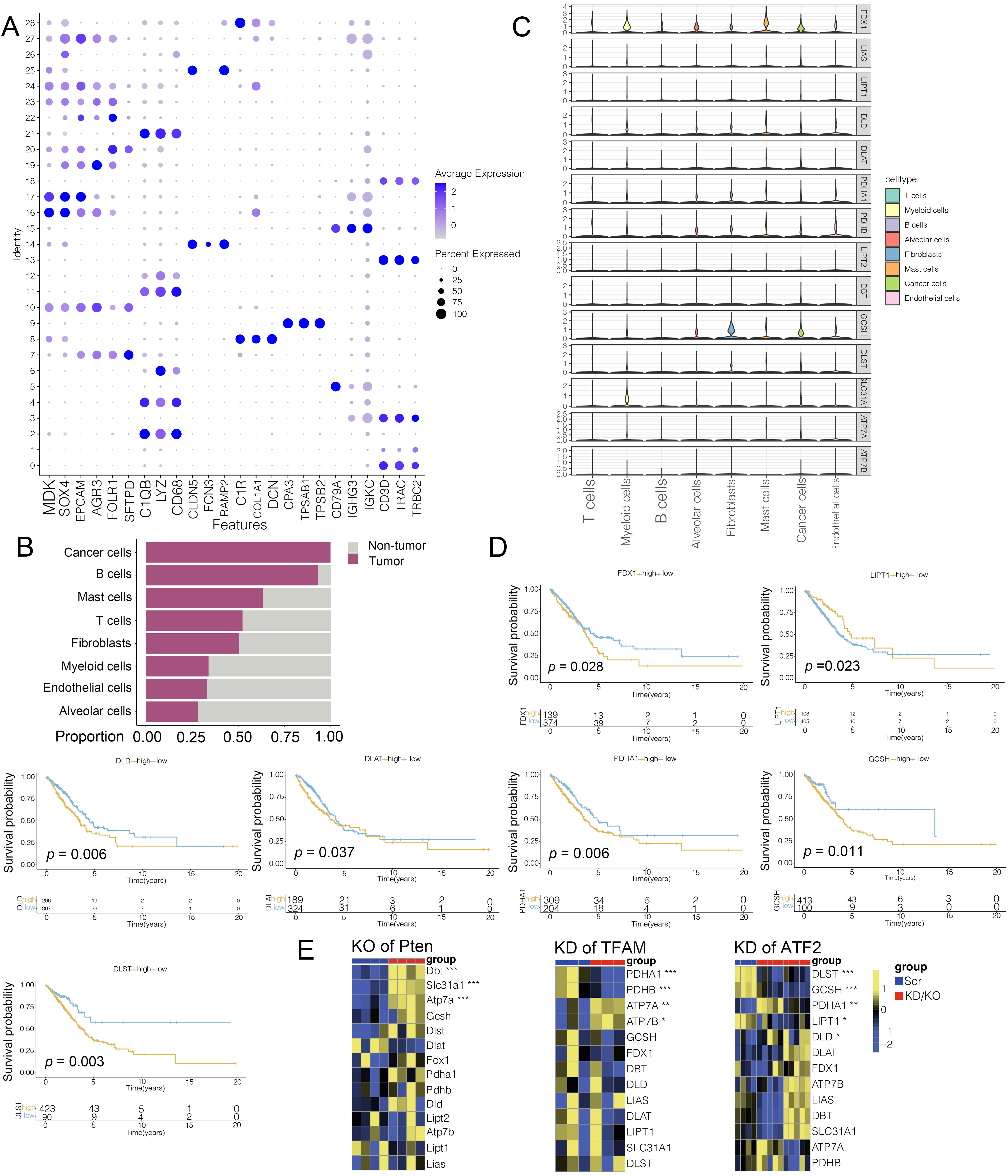

Supplement: Supplementary file 3 [file Image_2.tif]

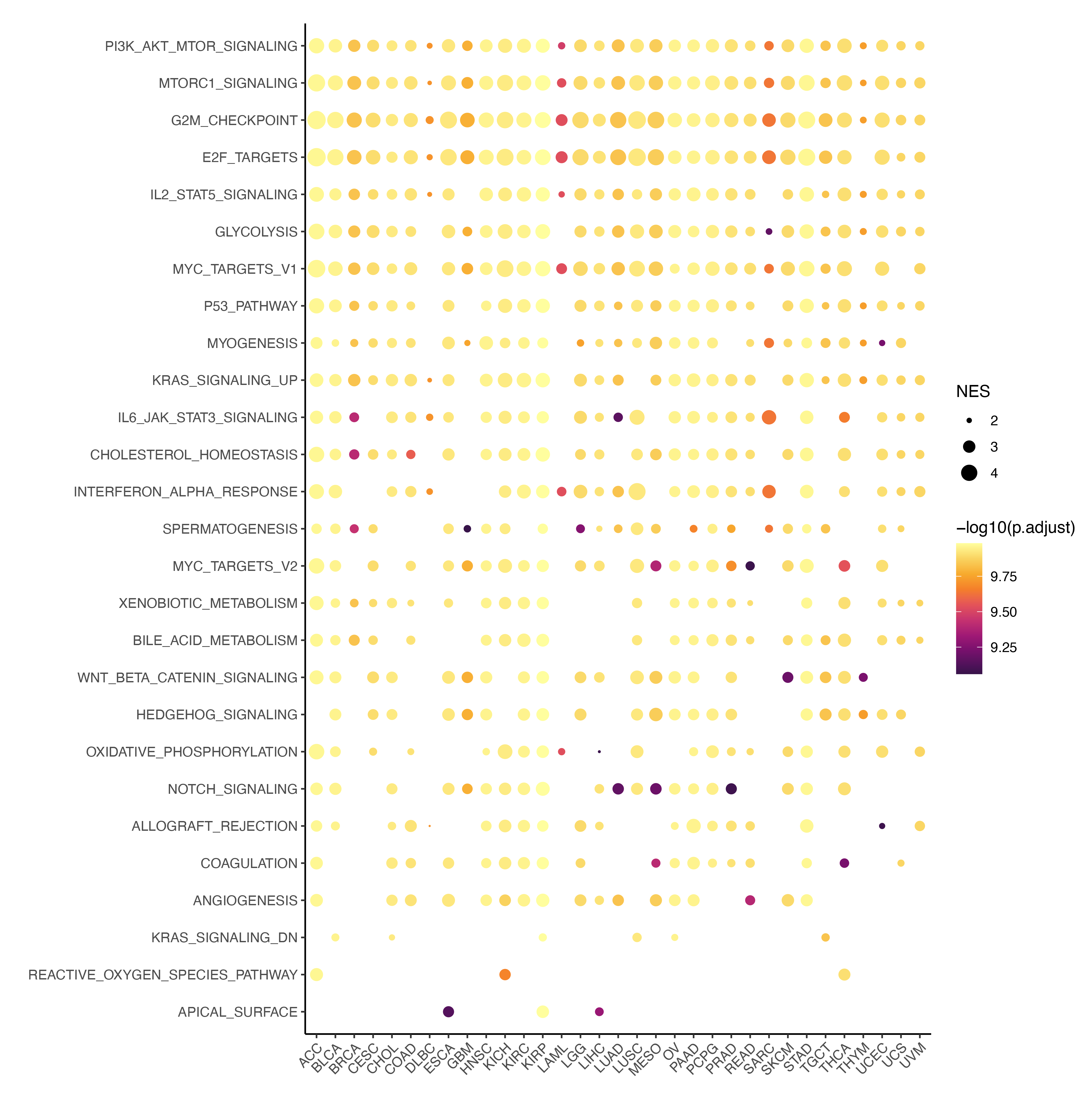

Supplement: Supplementary file 4 [file Image_3.tif]

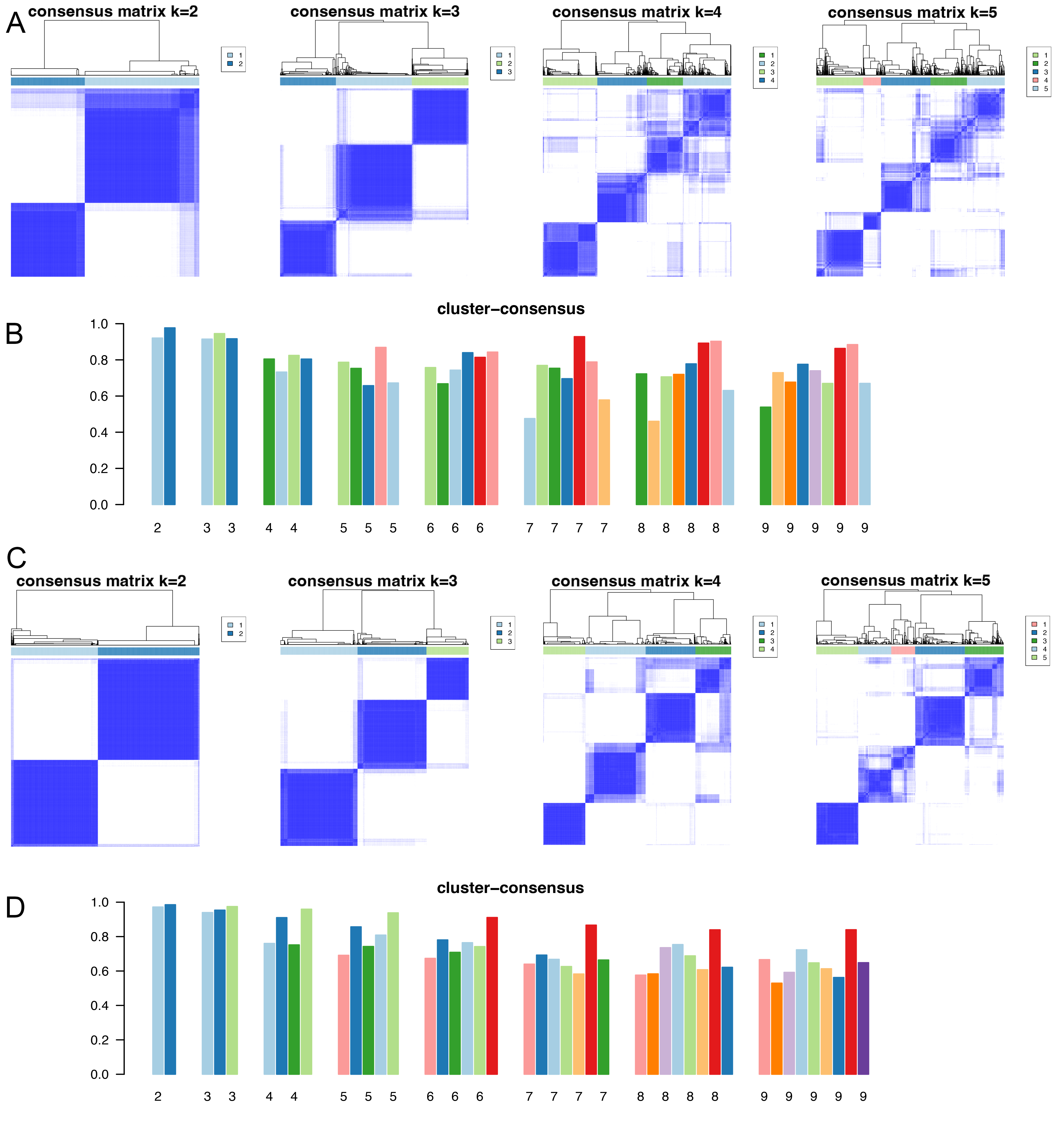

Supplement: Supplementary file 5 [file Image_4.tif]

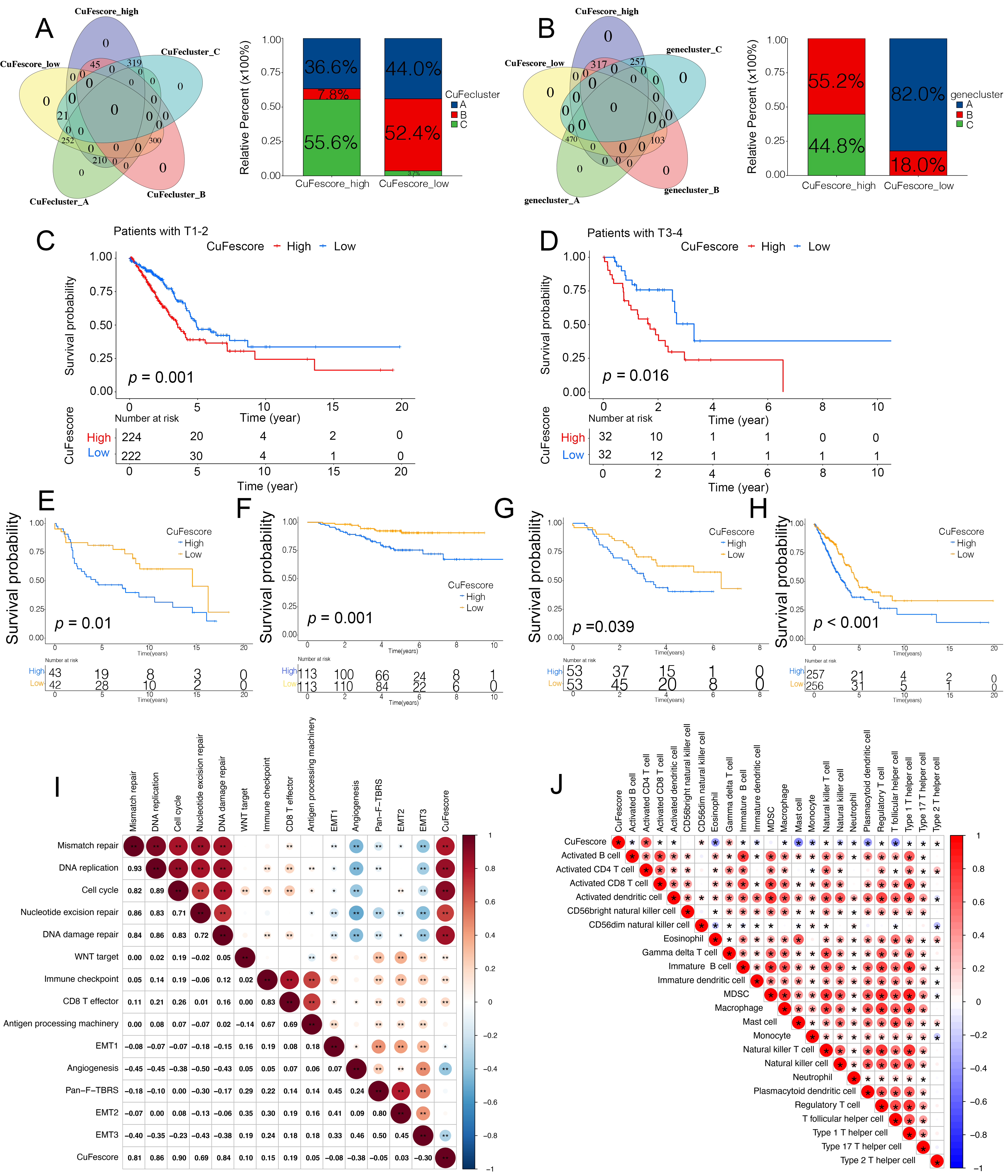

Supplement: Supplementary file 6 [file Image_5.tif]
